# Developing an App for Real-Time Daily Life Observations in a Nursing Home Setting: Qualitative User-Centered Co-Design Approach

**DOI:** 10.2196/57911

**Published:** 2025-02-27

**Authors:** Coen Hacking, Bram de Boer, Hilde Verbeek, Jan Hamers, Sil Aarts

**Affiliations:** 1 Department of Health Services Research, Maastricht University, Duboisdomein 30, Maastricht, 6229 GT, the Netherlands, 31 43 38 81570

**Keywords:** co-design, user-centered design, app development, nursing home, user-centered, design, efficiency, usability, tablet, mobile phone

## Abstract

**Background:**

Assessing the daily lives of older adults, including their activities, social interactions, and well-being is essential, particularly in nursing homes, as it gains insights into their quality of life. Methods such as the Microsoft Excel-based Maastricht Electronic Daily Life Observation (MEDLO) tool are time-consuming and require extensive manual input, making them difficult to use.

**Objective:**

This study aimed to develop an app-based version of the MEDLO using a user-centered design (UCD) and co-design approach to enhance efficiency and usability. We looked to actively involve researchers and care professionals who have used the MEDLO before, throughout the development process.

**Methods:**

Participants included a diverse group of researchers and care professionals experienced in using the MEDLO tool. The UCD approach involved multiple iterative phases including semistructured interviews, user research sessions, and application development. Data were analyzed using a qualitative (thematic) approach of UCD and user research sessions. The app, which was preferred to the traditional Excel-based MEDLO, underwent multiple iterations. This method primed the continuous iterative development of the app, aimed for a minimum viable product (MVP).

**Results:**

This study included 14 participants, primarily female, from diverse professional backgrounds. Their feedback highlighted the need for efficiency improvements in tool preparation and data management. Key improvements included automated data handling, an intuitive tablet interface, and functionalities such as randomization and offline data syncing.

**Conclusions:**

The iterative development process led to an app that aligns with end-user needs, indicating potential for improved usability. Early and continuous user involvement was key in enhancing the application’s usability, demonstrating the importance of user feedback in the development process.

## Introduction

Assessing the daily lives of older adults, particularly in the context of nursing homes, is essential for gaining insights into quality of life [1,2]. It allows health care providers to detect and address issues related to their physical health (eg, mobility limitations, pain), mental well-being (eg, mood disturbances, cognitive decline), and social interactions (eg, isolation, engagement in activities) in a timely manner [1,3,4]. This information can then be used to personalize care plans, thereby potentially improving health outcomes and enhancing the overall quality of life of older adults [5,6].

Ecological momentary assessments (EMAs) have been developed to facilitate this process by providing real-time, on-site evaluations of an individual’s well-being [5,7,8]. EMA involves collecting data on individuals’ behaviors and experiences in their natural environments, which can then be used to identify patterns and inform interventions [7]. However, existing EMA tools are mainly used in research settings, and their implementation in clinical practice has been challenging due to the time-consuming nature of data collection and the complexity of the tools [9,10]. The Maastricht Electronic Daily Life Observation tool (MEDLO-tool) is designed to assess the daily lives of nursing home residents using EMA methodologies [1,4]. The tool captures real-time information across several key dimensions of daily life, including activity levels (eg, participation in communal or solitary activities), agitation, mood states, and interactions with staff, fellow residents, and visitors [1,4,11]. By systematically observing and recording these aspects, the MEDLO-tool provides a nuanced view of residents’ experiences, which can inform personalized care strategies [4]. However, the MEDLO-tool relies on Excel templates that require significant manual input and are not user-friendly for care professionals [1,4]. The complexity of the Excel-based system poses significant challenges, including data entry errors, time inefficiency, and barriers to widespread adoption in clinical settings [12,13]. This complexity can be particularly problematic in nursing homes, where staff may have limited time and technological proficiency [13].

Developing a mobile application for the MEDLO-tool could address these challenges by automating data collection processes, reducing manual input, and providing an intuitive interface for users [14,15]. An app could streamline observations, enable real-time data analysis, and facilitate immediate feedback to care providers, thereby enhancing the tool’s use in clinical practice. Furthermore, considering the unique needs of residents with dementia, it is important that such an app is designed to account for cognitive impairments and communication difficulties [16,17]. The development of such an app requires a thorough understanding of the needs and preferences of the end-users. A user-centered design (UCD) approach is vital for developing software that truly meets users’ needs [14,18]. UCD emphasizes involving users throughout the design process to ensure that the product aligns with their requirements and preferences [19]. This involves iterative cycles of user need assessment, design, prototyping, and testing [20]. Co-design, a component of UCD, involves direct collaboration between users and designers, requiring the active participation of users in generating ideas, making decisions, and solving problems [21,22]. This not only ensures the software’s functionality but also its usability [21].

Potential barriers to implementing such technology include technological literacy among staff, user engagement, and ensuring data privacy and security [13,23]. By actively involving care professionals and stakeholders in the design process, we aim to create a tool that is both functional and user-friendly, facilitating its adoption in clinical practice and ultimately improving the quality of care for residents with dementia. Therefore, this study is aimed at developing an app for measuring the daily life of residents with dementia living in nursing homes, using a user-centered and co-design approach.

## Methods

### Study Design

**Figure 1.**
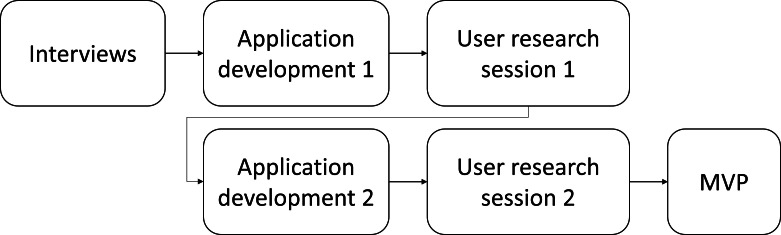
The iterative process of app development. MVP: minimum viable product.

This methodological study aimed to develop a mobile application version of the MEDLO-tool for use in long-term care facilities, using a UCD approach [24-26]. The study unfolded in multiple iterative phases, including semistructured interviews, user research sessions, application development, and comprehensive data analysis, as illustrated in Figure 1. The project culminated in the achievement of a minimum viable product (MVP).

### Participants

Participants were selected through purposive sampling to include individuals with experience using the MEDLO-tool. The sample consisted of researchers and health care professionals affiliated with Maastricht University and local nursing homes. Inclusion criteria were previous use of the MEDLO-tool and willingness to participate in the study. A total of 14 participants were recruited, comprising research assistants, PhD candidates, postdoctoral researchers, senior researchers, occupational therapists, research coordinators, and associate professors.

### Data Collection

Data collection involved semistructured interviews and user research sessions conducted between January and June 2023.

#### Interviews

Semistructured interviews were guided by a comprehensive list of topics derived from available literature and consultation with the researchers who developed the original MEDLO-tool [1,4]. The interview guide can be found in Multimedia Appendix 1. The interviews aimed to gather insights into participants’ experiences with the Excel-based MEDLO-tool, usability issues, functionality requirements, data analysis techniques, and general impressions [27]. Each interview lasted approximately 45 to 60 minutes and was audio-recorded with participants’ consent. The audio was transcribed verbatim for analysis. The interview guide is provided in the supplementary materials.

The interview transcriptions were subjected to a 6-phase thematic analysis process, aligning with the methodologies supported by previous research [28-30]. The inductive analysis was conducted by one researcher. A second researcher reviewed the coding to enhance trustworthiness. This comprehensive process involved familiarization with the data, generating initial codes, identifying themes, reviewing themes, defining and naming themes, and ultimately, reporting the results. The resulting report summarized the key findings, interpretations, and implications, to provide valuable insights for the next stages of the development of the app.

#### User Research Sessions

A total of 2 user research sessions were conducted monthly to involve participants in the iterative development process. The session guide can be found in Multimedia Appendix 2. Each session lasted approximately 2 hours and was held at Maastricht University. Participants interacted with app prototypes using tablets and smartphones provided by the research team. The sessions included guided tasks (eg, completing an observation using the app), open-ended discussions, and interactive feedback activities facilitated by researchers using prototypes and interactive demos. The structure of these sessions was informed by previous co-design methodologies [1,3,31].

### Data Analysis

#### Qualitative Analysis of Interviews

Interview recordings were transcribed verbatim and analyzed using thematic analysis following Braun and Clarke’s 6-phase approach [28]. A total of 2 researchers independently coded the transcripts to enhance reliability. Initial codes were generated and organized into potential themes. Discrepancies in coding were discussed and resolved through consensus meetings. Themes were reviewed and refined to ensure they accurately represented the data. The final themes captured key insights into the usability and functionality of the MEDLO-tool, informing the app development. The overall process of thematic analysis was guided by earlier works in this field [29,30]. The session guide is provided in Multimedia Appendix 1.

#### User Research Sessions

Each user research session included guided tasks (eg, completing an observation using the app), open-ended discussions, and interactive feedback activities facilitated by researchers using mock-ups and interactive demos. Feedback from user research sessions was documented through field notes and audio recordings. An inductive content analysis was conducted by one researcher, who coded feedback and categorized issues into 4 themes: layout, functionality, errors, and appearance. A second researcher reviewed the coding to enhance trustworthiness. This categorization allowed the development team to prioritize user concerns based on frequency and severity, ensuring a user-centric approach to app refinement.

### Application Development

The application was developed using “.NET MAUI” (ie, a framework designed by Microsoft) for cross-platform compatibility on iOS and Android devices. The server-side (ie, where the data are sent to) used “ASP.NET Core” (ie, a framework designed by Microsoft) for a maintainable and performant application. The development process was iterative, with weekly meetings between developers and researchers to integrate user feedback from the interviews and user research sessions.

Usability testing involved participants completing specific tasks using the app prototypes while researchers observed and noted any issues. Testing sessions were conducted in a controlled environment to simulate actual usage conditions. Feedback from these tests informed further refinements and addressed potential adoption or feasibility issues.

### Ethical Considerations

This study did not require formal ethics approval under the Medical Research Involving Human Subjects Act (WMO). In the Netherlands, research that collects only anonymized or nonsensitive feedback—without requiring participants to undergo procedures that affect their physical or psychological integrity—is not considered WMO research and is therefore exempt from ethics review. The guidelines provided by the Central Committee on Research Involving Human Subjects clarify that retrospective or noninvasive studies using aggregated or anonymized data do not fall under the scope of the WMO [32]. The Maastricht University Medical Center+ Research Code also confirms that studies conducted with minimal risk, such as those analyzing collective feedback, do not require formal ethical review. All participant feedback was anonymized and is presented in aggregate form to ensure confidentiality [33].

## Results

### Sample

The study involved 14 participants with an average age of 36 (SD 10; median 39; range 24‐57) years, and 93% (13/14) of them were female. The participants represented a diverse range of professions, including research assistants (n=3), PhD candidates (n=4), postdoctoral researchers (n=2), a senior researcher (n=1), an occupational therapist (n=1), a research coordinator (n=1), an associate professor (n=1), and an individual who was currently unemployed. Most participants (9/14, 64%) were affiliated with Maastricht University, while others worked at health care providers (4/14, 29%) or were unemployed (1/14, 7%). The average duration of employment at their respective institutions was 6 (SD 5; median 6; range 0.5‐16) years.

All 14 participants had previous experience with the MEDLO-tool; 12 (86%) participants had used it directly in their research, while 2 (14%) participants had used it as inspiration for developing other tools different from MEDLO. All participants had also been in contact with the developers of the MEDLO-tool. Table 1 presents the demographic information of the participants in detail.

**Table 1.** Demographic information of the participants.

Characteristic		Values
Age (years), mean (SD)		
	24‐57	36 (7.7)
Gender, n (%)		
	Female	13 (93)
	Male	1 (7)
Time at institution, mean (SD)		
	6 months to 16 years	6 (3.3)
Use of the tool, n (%)		
	Used in research	12 (86)
	Inspiration for another tool	2 (14)
Contact with developers, n (%)		
	All participants	14 (100)
Participants’ affiliations, n (%)		
	University	9 (64)
	Health care provider	4 (29)
	Unemployed	1 (7)

### Interviews

The participants described the process of using the MEDLO-tool in research projects as comprising 7 distinct phases: (1) acquiring informed consent, (2) preparing the Excel sheets before the observation, (3) familiarizing themselves with the faces of the residents who would be observed, (4) conducting the observations, (5) fixing issues with the data (such as missing values or inconsistencies), (6) analyzing the data, and (7) communicating the data back to the care organizations.

Participants emphasized that the preparation of the tool and the subsequent data cleaning were particularly time-consuming and would significantly benefit from automation. One participant stated, “Every time before we do a new observation, we have to look at the participants, randomize them several times, and input all of this into the Excel sheet.” Another participant highlighted the challenges faced after data collection, *“*After the observation is done, I have to go through the whole document to check all the fields that I didn’t have time to fill out or that I didn’t know how to rate.” These comments reflect the manual and labor-intensive nature of the existing process, which participants found cumbersome.

Participants reported that during observations, they used separate pieces of paper to write down descriptions of each resident’s appearance because the observation periods were too short to input this information into the Excel sheet in real time. They suggested that having an additional field to securely write this information directly on the tablet would make the process more accessible. One participant explained, “We usually arrive at least half an hour earlier, so we can ask the nurses which participant corresponds to which name, and write down what they look like. We do that to find them back more easily during the observation.” Another participant elaborated on the logistical challenges, “The data entry process on the tablet is intuitive, but we also have to keep track of the patient names and descriptions on paper, we carry the manual, and we use a timer on our phone to ensure that we don’t spend too long on any resident. This is a lot to carry around.” Regarding data reporting, participants indicated that the data reported back to the nursing homes primarily consisted of quantitative measures, which could potentially be automated through a dashboard (eg, Microsoft PowerBI). Participants expressed that automating this process would reduce the time between data collection and reporting, which currently could be several months. One participant noted, “When we want to report back the numbers to the care organization, we make a PowerPoint presentation that includes aggregated numbers. This usually takes place several months after the observations.” Another participant suggested: “I think some of the numbers could be reported back automatically.”

Overall, participants preferred using a tablet compared to using pen and paper, citing the intuitive data entry process. However, they also mentioned that a tool compatible with smartphones would be beneficial, especially for care professionals who might find tablets less convenient. One participant remarked, “I think the tool works well, but I’m not sure if it would be usable like this for nurses.”

### Initial User Research Session

Based on the information gathered during the interviews and the suggested points for improvement, an initial version of the application was created and provided to the participants for the first user research session. This first draft of the application consisted of an dashboard web application and a mobile app, both of which included the core components but had limited functionality. Multimedia Appendix 3 shows the app and dashboard as they were presented to the participants. Multimedia Appendix 4 provides a detailed overview the feedback received during the initial user research session.

During the initial user research session, participants expressed largely positive sentiments towards the concept of the application and appreciated the efforts made in its development. One participant commented, “I know we have a lot of complaints about the app, but we really appreciate the work [the development team] has done already.” However, participants also identified several areas for improvement. They expressed concerns regarding certain features of the app, such as the timer functionality, which lacked a reset option. Participants noted that in their current practice, they used interval timers on their phones to manage observation times for each resident, and the app’s timer did not adequately support this need. One participant suggested, “What could be nice is some kind of interval timer. That’s what we currently use on our phones.” In addition, participants missed the comprehensive overview provided by the Excel-based MEDLO-tool, as the prototype app had been designed primarily for phone screens and did not offer the same level of data visibility. One participant observed, “Normally, you could easily scroll back in the overview.” Furthermore, there were some unclear aspects of the app, such as certain functionalities being available only through the dashboard and not within the app itself. Participants found labels on buttons to be unclear, which led to confusion during navigation. They recommended improving the screen layout for better usability and enabling easy switching between participants by a list.

Participants raised concerns about data storage and security, even though these aspects had been addressed in the app’s design. They questioned how the data was stored and whether it was encrypted. It was explained to them that the data was stored on the device (ie, phone or tablet) in encrypted form and could be synced to a secure server. Regarding randomization, participants asked how it would be conducted within the app and where they could access this feature. It was clarified that randomization was performed automatically upon creating a new observation group. A participant inquired, “How do I randomize the participants in the app?” To which the response was, “That is done automatically when an observation group is added.”

Participants inquired about features that had not yet been implemented but were planned for future updates. For example, they asked if the app would work offline, which was an important consideration for use in environments with limited internet connectivity. They also asked whether the data could be exported to Excel for further analysis. These features were already part of the application’s development roadmap but were not yet available in the user interface at the time of the session. One participant expressed concern, “I don’t know if it’s an issue when I lose connection, or whether I lose my data.” Another participant discussed data export capabilities, “These are the data you see when you download the Excel file. With Excel, it looks a bit different, but what you see here are all the column names.”

### Second User Research Session

Following the initial user research session, the app was updated to address the feedback received. Adjustments were made to the timer functionality, screen layout, language translation, and several other features as per the participants’ suggestions. A second version of the application was then provided to the participants for the subsequent user research session. In Multimedia Appendix 5, the feedback received during the second user research session is detailed.

In the second session, participants expressed fewer concerns regarding the application, indicating that many of their initial issues had been resolved. However, they still provided valuable feedback on functionality and appearance. One of the main concerns was that the app was not yet fully translated into Dutch; while significant progress had been made, some parts remained in English. Participants emphasized the importance of complete translation before the app could be effectively used by nurses and other care professionals. One participant remarked, “Most of the app has been translated, but some of the texts are still only in English.” Another participant added, “This all needs to be translated before we can hand this to nurses.”

Participants also suggested that the criteria for showing and hiding fields during observations should be further refined based on relevance, to streamline the data entry process and reduce cognitive load. They discussed various small changes in the appearance of the app to enhance usability and visual appeal. For instance, they recommended that the “star” button used to mark important observations be made a different color to stand out more prominently. One participant suggested, “Could you make the star button orange to make it stand out a bit more?” In addition, participants found the criteria for marking an observation as “complete” to be somewhat confusing. They proposed that once an activity has been set for an observation, it should be automatically marked as complete to provide clearer feedback to the user. One participant explained, “I would prefer it if the observation could be marked as ‘complete’ when the activity has been set.” Participants also recommended offering flexibility in the number of observation rounds that could be added, as different research protocols or care routines might require varying numbers of observations. They suggested making the process more efficient by creating a priority list or extending the default time block for certain observations that typically take longer. Furthermore, participants emphasized the importance of ensuring that the app’s manual could be accessed from within the app itself. They stressed that the manual should be clear, comprehensive, and align with users’ practical experiences to facilitate ease of use, especially for new users.

By the conclusion of the second user research session, participants expressed optimism about the app’s potential to improve their workflow and reduce the time spent on manual data entry and processing. They acknowledged the responsiveness of the development team to their feedback and looked forward to future iterations of the app that would incorporate their latest suggestions.

## Discussion

### Principal Findings

This study successfully developed an app-based version of the MEDLO-tool aimed at assessing the daily life of residents with dementia in nursing homes. By using a UCD approach, we incorporated feedback from participants throughout the development process, resulting in a minimum viable product that aligns with the needs and preferences of end-users.

The iterative development process revealed that larger issues, such as bugs and significant feature requests, were identified and addressed in the initial phases. Subsequent user research sessions showed a decrease in the number and severity of issues, indicating progressive refinement of the app. Participants expressed that the app improved upon the Excel-based MEDLO-tool by streamlining data collection and reducing manual input, thereby potentially enhancing usability.

Balancing end-user feedback with established best practices and user interface guidelines was crucial during development [34-37]. While participants provided valuable suggestions, not all could be implemented due to conflicting requests and constraints related to usability standards. For instance, some users desired a more complex timer feature, whereas others preferred simplicity. Decisions were made to benefit the majority and adhere to platform guidelines to ensure familiarity and ease of use [34,35].

The effectiveness of the UCD approach in this study aligns with previous research demonstrating its benefits in software development [38,39]. A key factor in our success was the development team’s familiarity with the context of long-term care for older adults. This domain knowledge facilitated insightful conversations between developers and participants, reducing misunderstandings and accelerating the development process. Previous studies have highlighted that cognitive similarity and shared jargon can enhance team performance [40,41].

### Limitations

Despite these strengths, the study has limitations that warrant consideration. The participant sample consisted primarily of researchers affiliated with the University, which may limit the generalizability of the findings to care professionals who are the intended end-users in clinical settings. While these researchers had extensive experience with the MEDLO-tool, involving a broader range of stakeholders, including nurses and other care staff, could provide additional insights into usability and practical implementation. Furthermore, the app was not tested in a real-life nursing home environment, which could have revealed context-specific challenges and opportunities.

### Future Work

Future research should focus on conducting pilot studies to evaluate the feasibility and effectiveness of the MEDLO app in real-world nursing home settings. Involving care professionals in these studies would help assess the app’s usability, accessibility, and impact on daily workflows. In addition, gathering feedback from residents and their families could provide a more comprehensive understanding of the app’s influence on care quality.

Further development could explore integrating advanced technologies, such as artificial intelligence and machine learning, to enhance data analysis and provide predictive insights [42,43]. For example, natural language processing could be used to automate the interpretation of observational data, aiding care providers in identifying patterns and potential areas for intervention. However, implementing such technologies would require careful consideration of ethical implications, data privacy, and user acceptance.

Future research may also investigate the usability in practice, by letting nurses use the app in their daily work. This could provide valuable insights into the app’s integration into existing workflows and its impact on the quality of care provided to residents. In addition, longitudinal studies could assess the long-term effects of using the app on care outcomes and staff satisfaction. This information could inform further refinements and improvements to the MEDLO app, ensuring its relevance in long-term care.

### Conclusion

This study successfully demonstrates the viability and demand for an app-based MEDLO-tool for assessing residents with dementia in nursing homes. By using a UCD approach, this study addressed the limitations of the existing Excel-based system by offering a more efficient and user-friendly alternative. This study shows that involving users early on in the process and keeping them involved can have a positive effect on the usability of an application. The MEDLO app shows that a UCD approach can provide real benefits in the development of a digital tools in nursing homes.

## Supplementary material

10.2196/57911 Multimedia Appendix 1 The interview guide used to the conduct the initial interviews.

10.2196/57911 Multimedia Appendix 2 The interview guide used for the user research sessions.

10.2196/57911 Multimedia Appendix 3 A screenshot of what the dashboard of the application looks like.

10.2196/57911 Multimedia Appendix 4 Feedback from the initial user research session.

10.2196/57911 Multimedia Appendix 5 Feedback from the second user research session.

## Abbreviations

EMA: ecological momentary assessment
MEDLO: Maastricht Electronic Daily Life Observation
MEDLO-tool: Maastricht Electronic Daily Life Observation tool
MVP: minimum viable product
UCD: user-centered design
WMO: Medical Research Involving Human Subjects Act
