# Supplementary material for: Developing an App for Real-Time Daily Life Observations in a Nursing Home Setting: Qualitative User-Centered Co-Design Approach
Source: JMIR Hum Factors. 2025 Feb 27;12:e57911. doi: 10.2196/57911 (PMC11884308; doi:10.2196/57911)
Supplement: Multimedia Appendix 1 [file humanfactors-v12-e57911-s001.docx]

# MEDLO-tool interview questions

## Interviewee information

1) What is your age?

2) Where do you work?

3) What is your title/function/job?

4) How long have you worked in this position?

5) What is your general area of research/work?

## Interviewee Research Details

6) How did you find out about the Maastricht Electronic Daily Life Observation (MEDLO) tool?

7) When did you use the MEDLO-tool?

8) Did you have contact with the developers of the MEDLO-tool prior to using it? If so, what was discussed?

9) What were you investigating when using the tool? (Aim, length of study, number of observations).

10) How did you prepare to use the tool? Did you read the manual before your study? Was it clear? Did it sufficiently inform you of the tool?

11) Did you follow the plan the manual advised? (Several mornings, afternoons and evenings or another plan).

12) Did you make any adjustments to the MEDLO-tool before use in your study?

## Use of the MEDLO-tool

13) Was the MEDLO-tool easy/difficult to use? Particularly, on first use and then after some time.

14) Were the domains of daily life easy to understand?

a) Do you believe that the four domains of daily life cover all aspects of daily life (Activities, Physical Location, Social Interaction, Emotional Well-Being)?

b) Were the options available for each domain in the drop-down menus easy to understand? Does it sufficiently cover all aspects? Anything missing or superfluous?

## Analysis

15) After your observation sessions, how did you analyse the data? Did you use excel, SPSS or another program?

a) If SPSS: Was it easy to upload your data to SPSS? Any issues/problems?

16) What were your experiences with the analysis of the data collected by the MEDLO-tool? Any issues/problems with the analysis?

## Opinion

17) What were your overall experiences with the MEDLO-tool?

18) Were there aspects of the MEDLO-tool that you liked or that worked very well?

19) Were there aspects of the MEDLO-tool that you struggled with or did not work well?

20) Are there any improvements you think should be made?

## General Remarks

21) Would you use the tool again?

22) Do you know of anyone else who has used the tool?

23) Do you have any other comments about the MEDLO-tool?

24) Have you used any other observational tools/methods/etc.?
