# Supplementary material for: Developing an App for Real-Time Daily Life Observations in a Nursing Home Setting: Qualitative User-Centered Co-Design Approach
Source: JMIR Hum Factors. 2025 Feb 27;12:e57911. doi: 10.2196/57911 (PMC11884308; doi:10.2196/57911)
Supplement: Multimedia Appendix 2 [file humanfactors-v12-e57911-s002.docx]

# Welcome (2 minutes)

Welcome to this user research session. Today, we are here to discuss the MEDLO app, its design, functionality, and potential for integration into your workflows and the workflows of nurses. Your input and opinions are greatly valued, as they will help shape the future development of the MEDLO app and enable us to make improvements that are specifically tailored to your needs and expectations.

As we delve into the topics, feel free to share your thoughts, opinions, and concerns, even if they differ from those of others. All viewpoints are welcome, and they will be considered during the development of the MEDLO app.

I will serve as the moderator of this discussion, keeping us on track and managing the time allocated for each topic. If you have any questions or concerns, please do not hesitate to ask me. Additionally, Sil will be taking minutes for this session, and Bram will help keep the focus during the discussion. I will be using a laptop to refer to the questions, take a few notes, and show examples of the user interface during the session.

Once again, thank you for your participation, and I look forward to hearing your valuable insights.

# Anonymity and Confidentiality

Your privacy is important to us. All information you share during this session will be kept confidential and anonymous. We will not share your personal information or any identifying details with anyone outside of our research team.

# Audio Recordings

We will be recording this session for research purposes. The recordings will only be used internally to help us better understand our users and improve our app. They will be kept confidential and not shared outside of our research team. I will now ask you to sign the informed consent form so that we can start the audio recording.

# Code of Conduct

To ensure a respectful and productive discussion, it is important to follow a few basic rules. Firstly, let’s listen actively to each other and avoid side conversations. Let’s also allow everyone to finish speaking and make sure we interact respectfully. Finally, to maintain confidentiality, I ask you not to quote or attribute others’ comments outside of this discussion.

# Oral Summary

As we go through each topic during this session, we will take a moment to summarize briefly. This will give us the opportunity to ensure that we have captured all the important points and provide you with the chance to add or clarify anything you feel has not been fully addressed. This way, we can accurately capture each theme and viewpoint expressed, explore the implications, and generate ideas on how to use this input to improve the MEDLO app further.

# App Information

For this session, you have already had access to the MEDLO app prototype to try out. If you haven’t had a chance to look at it yet, that’s okay. I will now give a demonstration of the current prototype of the MEDLO app to provide you with an overview of its design, functionality, and usability. If any part of this demonstration is unclear, feel free to ask questions. This will ensure that the subsequent discussions are as valuable as possible.

# Interview Questions

## Initial User Experience

This topic focuses on how easy or difficult it is for a first-time user to use the prototype app. The questions are intended to identify any challenges or ease of use that the user experienced while navigating the app, as well as any confusing or intuitive aspects of the app.

| **Question** | **Follow-up Questions** |
| --- | --- |
| What is your first impression? |  |
| Was it clear what all the buttons and labels meant? | What things were unclear? |
| Can you describe any issues or ease you experienced when navigating the app? | Were there specific areas in the app where you struggled with navigation? Were there parts of the app that were particularly easy to navigate? |
| How difficult is it to learn how to use the app? | Can you point out specific features that were hard or easy to learn? Did you need help or additional information to understand or use certain features? Can you explain why you found it more or less difficult to learn? |
| Were the instructions and guidance in the app sufficient to learn how to use it? | What was unclear? Can you provide examples? |
| How satisfied are you with the overall speed of the prototype? | Did you notice any lag or delay while using the app? How did the app’s speed affect your experience? |
| Were there any other things that stood out to you? |  |
| Are there things that are now clear after the brief demonstration of the app that were unclear before? |  |

## Visual Design

This topic focuses on the visual design of the prototype app. The questions are aimed at identifying aspects of the design that are visually appealing or unappealing and gaining insights into why the user perceives them that way.

| **Question** | **Follow-up Questions** |
| --- | --- |
| What are your first impressions of the design of the prototype? | What aspects caught your attention first? Did you feel drawn to the design? If so, why? |
| How visually appealing is the prototype app? | Is there a specific part of the app that you find particularly visually appealing? Are there any aspects of the app that you would describe as visually unappealing or distracting? |
| Is the layout and organization of information in the app intuitive? | What aspects of the layout and organization caught your eye first? Were there any parts of the app that you found confusing or unnecessarily complicated? Were you able to find what you were looking for easily? |
| Can you give examples of specific aspects of the design that you liked or disliked? | What appeals to you the most about the current design, and why? Are there any design elements that distract or irritate you? If so, which ones and why? If you could change one aspect of the design, what would it be? |
| What do you think of the color scheme? | Do you find the colors attractive? Do you think the color choice contributes to the overall aesthetic of the app? If so, how? |
| What do you think of the font size? | Is the font size comfortable for you to read? Do you think the font size is appropriate for the app’s content? |
| What do you think of the icons? | Do the icons match the text? Where are the icons unclear? |

## Functionality

This topic focuses on the features of the prototype app. The questions are aimed at identifying whether the app addresses the challenges and limitations of the previous tool or process, as well as identifying any missing features or features the user would like to add.

| **Question** | **Follow-up Questions** |
| --- | --- |
| Is the app functionally comparable to the Excel-based tool? Or better, or worse? | Can you give specific examples of the challenges you had with the Excel-based tool and how the app addresses these? Are there challenges that have not yet been addressed? If so, which ones? |
| Can you give specific examples of how the app addresses these challenges? | What are some new features or design elements that have solved these problems? Are there elements that do not work quite the way you would like? If so, how could they be improved? |
| Are any features or functions missing? If so, can you give examples? | What features would you like to see added? How would these features improve the user experience? What functionalities do you miss the most? Have there been new challenges due to using an app? |

## Integration into the Workflow

This topic focuses on whether the prototype app fits into the user’s workflow, either as an individual user or as part of a healthcare professional’s workflow. The questions are intended to identify any changes that would be needed to integrate the app into the user’s existing workflow.

| **Question** | **Follow-up Questions** |
| --- | --- |
| Do you think the app could fit into a nurse’s workflow in its current form? | Why or why not? Can you name specific tasks or processes where you would find the app useful? |
| Can you imagine using the app in a healthcare professional’s workflow? | How do you think the app could support a healthcare professional’s daily tasks and responsibilities? Are there aspects of the app that might be less relevant or useful in this context? |
| What changes, if any, would you need to make to your workflow to use the app? | Are there specific adjustments you would need to make to your current tasks, processes, or schedule to use the app efficiently? If so, what are they and why? |

## Privacy

This topic focuses on the privacy implications of using the prototype app compared to the previous tool or process. The questions are aimed at identifying whether the app offers better, equal, or worse privacy than the previous tool or process.

| **Question** | **Follow-up Questions** |
| --- | --- |
| At the moment, some of you have mentioned using a separate sheet with the names and numbers of participants during interviews. Do you think this app improves privacy? | Are there specific features or characteristics of the app that contribute to your perception of privacy? If so, what are they and how do they contribute to overall privacy? |
| Does the app offer equal, better, or worse privacy compared to the Excel-based approach? | On what aspects of privacy did you base this assessment? Do you think the app is generally more or less secure than the Excel-based approach? |
| Do you have privacy concerns when using the app? | Are there specific aspects of the app that concern you in terms of privacy? If so, what are they and why? |

## Dashboard Insights

This topic focuses on the descriptive data displayed on the dashboard after observations have been made.

| **Question** | **Follow-up Questions** |
| --- | --- |
| How clearly are the data displayed on the dashboard after making observations? | What elements of the data visualization did you find useful or confusing? What information was |

easy to interpret, and what information did you find difficult to understand? | | How useful did you find the insights displayed on the dashboard? | Were the insights displayed relevant to your needs? Which insights were most valuable and which were less useful? | | Was the information on the dashboard up to date? | Was the information displayed in line with your expectations in terms of timeliness? Did you notice any delay in updating the information on the dashboard after making an observation? | | How satisfied are you with the amount of information displayed on the dashboard? | Did you feel that the dashboard provided the right amount of information, or was it too overwhelming or too sparse? Are there specific data you would like to see on the dashboard that are not currently available? How can we improve the dashboard for practical use? |

# Conclusion

As we wrap up the session, I want to express my sincere thanks for your valuable input and the time you’ve spent sharing your opinions and experiences. Your feedback is invaluable to us, and we appreciate your efforts to help improve the MEDLO app. We will use the feedback gathered during this session to further develop and enhance the MEDLO app. I look forward to working with you again in the future. Thank you and goodbye.
