# Supplementary figures and images for: Developing an App for Real-Time Daily Life Observations in a Nursing Home Setting: Qualitative User-Centered Co-Design Approach
Source: JMIR Hum Factors. 2025 Feb 27;12:e57911. doi: 10.2196/57911 (PMC11884308; doi:10.2196/57911)

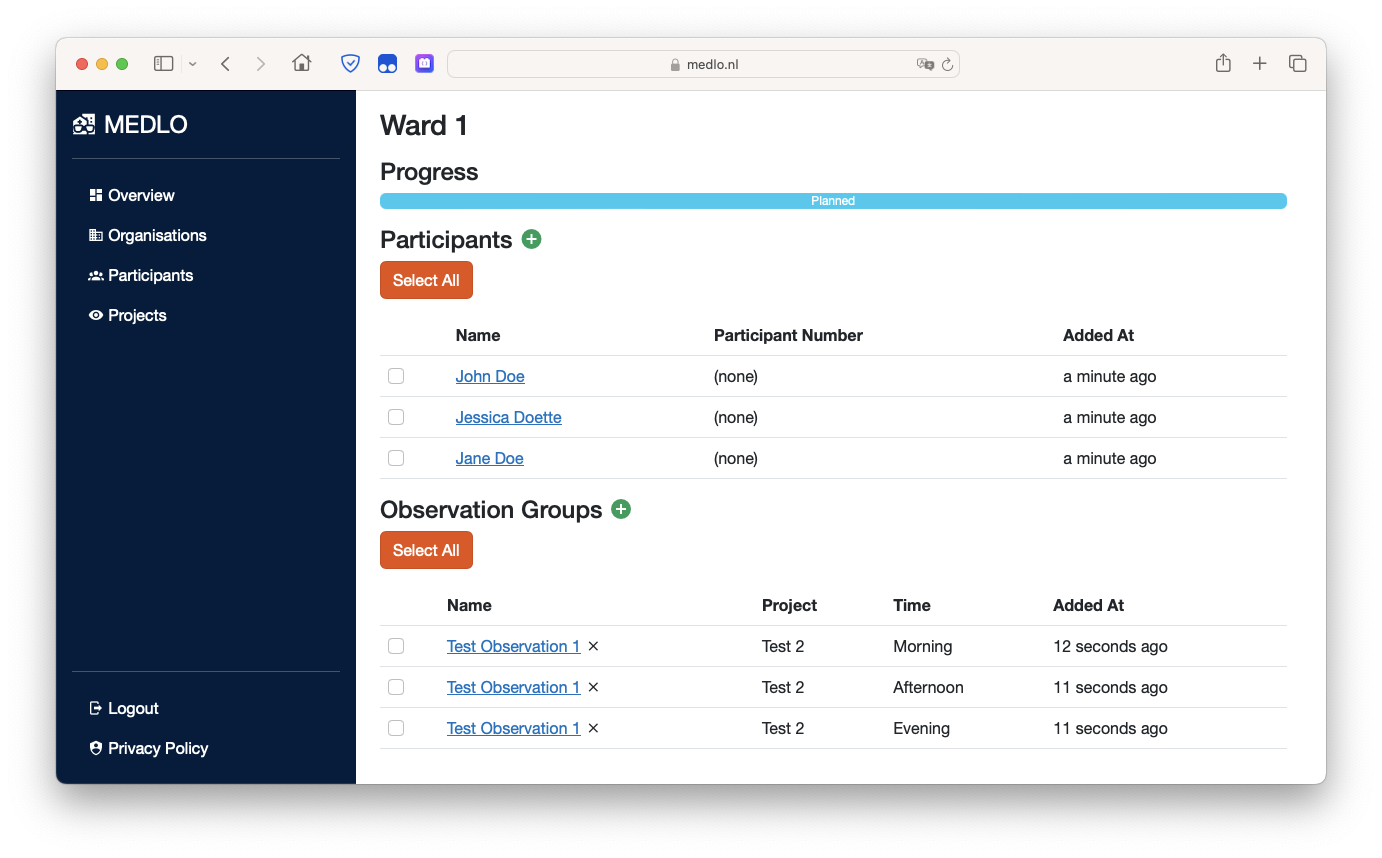

Supplement: Multimedia Appendix 3 [file humanfactors-v12-e57911-s003.png]
