# Supplementary material for: Developing an App for Real-Time Daily Life Observations in a Nursing Home Setting: Qualitative User-Centered Co-Design Approach
Source: JMIR Hum Factors. 2025 Feb 27;12:e57911. doi: 10.2196/57911 (PMC11884308; doi:10.2196/57911)
Supplement: Multimedia Appendix 4 [file humanfactors-v12-e57911-s004.docx]

**Table 2**: Feedback from the initial user research session.

| Type | Feedback | Addressed |
| --- | --- | --- |
| Functionality | Implement the functionality to search for participants and view projects. |  |
| Functionality | Adjust the timer to enhance its function. Currently, there’s no reset option. | Adjusted |
| Layout | Improve the screen layout for better overview and usability. Easy switching between participants via list. | Adjusted |
| Functionality | Allow ‘stars’ to be given to observations to easily find them later. | Adjusted |
| Functionality | Show an indicator an observation is completely filled in. | Adjusted |
| Functionality | Add the capability to save data and make it available for download. |  |
| Functionality | On disruption or sudden app closure, data should remain safe. The app should sync and work offline beforehand. |  |
| Functionality | Add a field for linking participant numbers in the app with numbers from other survey forms done in Excel. | Adjusted |
| Error | Update the app according to the latest MEDLO manual. Currently, there are inconsistencies in functions & terms. | Adjusted |
| Layout | Create a summary screen showing filled-in data so far. |  |
| Functionality | Implement the ability to view projects in the app. |  |
| Functionality | Add an option to upload a floor plan for easy location & participant identification. |  |
| Functionality | Ensure the app can be used entirely in Dutch. | Adjusted |
| Layout | Results should be more organized. There should be a filter option. Development over time isn’t important. |  |
| Appearance | “Morgen” isn’t immediately clear. “Ochtend” would be clearer. | Adjusted |
| Appearance | Colors need to be adjusted to match the AWO-L. | Adjusted |
