# Supplementary material for: Developing an App for Real-Time Daily Life Observations in a Nursing Home Setting: Qualitative User-Centered Co-Design Approach
Source: JMIR Hum Factors. 2025 Feb 27;12:e57911. doi: 10.2196/57911 (PMC11884308; doi:10.2196/57911)
Supplement: Multimedia Appendix 5 [file humanfactors-v12-e57911-s005.docx]

**Table 3**: Feedback from the second user research session.

| Type | Feedback |
| --- | --- |
| Functionality | While a portion of the app is translated, this process should continue to make all elements and features of the app fully available in Dutch. |
| Functionality | Consider adding a feature to remove participants, e.g. when a participant is deceased. |
| Functionality | The criteria for showing and hiding fields, based on what is relevant, during observation should be further extended. |
| Appearance | The criteria for marking an observation as ‘complete’ can be confusing. Once an observation has an activity, it should be marked as complete. |
| Appearance | Consider making the stars orange for improved readability and make the star button a different color. |
| Functionality | Offer flexibility in the number of observation rounds that can be added. |
| Functionality | Make the process more efficient by creating a priority list and/or extending the default time block for certain observations. |
| Functionality | Ensure that the manual can be accessed from within the app. It should be clear and comprehensive, aligning with users’ practice. |
